# Métastase cérébrale d'un carcinome du col utérin

**DOI:** 10.11604/pamj.2013.14.114.2215

**Published:** 2013-03-23

**Authors:** Tarik Chekrine, Abdesalam Hassouni, Hassan Jouhadi, Souha Sahraoui, Zineb Bouchbika, Amina Taleb, Nadia Benchakroun, Nezha Tawfiq, Abdellatif Benider

**Affiliations:** 1 Service de Radiothérapie-Oncologie, Centre hospitalier Ibn Rochd, Casablanca 1, quartier des hôpitaux, 20360 Casablanca, Maroc

**Keywords:** Métastases cérébrales, cancer du col utérin, traitement, pronostic, brain metastases, cervical cancer, treatment, prognosis

## Abstract

Les métastases cérébrales des cancers du col de l′utérus sont extrêmement rares. Elles sont généralement supra-tentorielles, survenant à un stade avancé de la maladie et dans un cadre de néoplasie polymétastatique. La tumeur primitive est le plus souvent un carcinome épidermoïde peu différencié. Leur pronostic reste sombre malgré toutes les options thérapeutiques. Vu la rareté de cet événement et le peu de cas publiés dans la littérature, nous rapportons l'observation clinique d'une jeune patiente de 44 ans, opérée pour un carcinome du col utérin et qui présente 14 mois plus tard des métastases cérébrales sus et sous tentorielles associées à des métastases ganglionnaires lombo-aortique, médiastinale et sus-claviculaire. Elle a bénéficié d'un traitement palliatif associant une chimiothérapie et une radiothérapie pan encéphalique. Devant l'altération rapide de l'état général, la patiente a été mise sous un traitement symptomatique et des soins de support.

## Introduction

Les métastases cérébrales au cours des cancers sont fréquentes. Leur fréquence dépend fortement de la tumeur primitive et de son histologie [1]. Les cancers primitifs les plus fréquemment à l′origine sont les cancers bronchiques, mammaires, colorectaux et rénaux [2]. Le cancer du col utérin est très rarement à l'origine de métastases intracrâniennes [3–5]. Nous en rapportons une nouvelle observation, une revue de littérature est aussi présentée.

## Patient et Observation

Une femme âgée de 44 ans, quatre pares et sans antécédents médicochirurgicaux particuliers, a consulté en Avril 2010 pour des leucorrhées blanchâtres associées à des métrorragies de faible abondance d'abord provoquées par les rapports sexuels puis spontanées. Ces symptômes évoluaient depuis six mois, sans douleurs pelviennes et sans troubles urinaires ni digestifs. L'examen clinique a trouvé une patiente en bon état général avec un performans status OMS à 0 et à l'examen gynécologique, une lésion ulcéro-végétante, hémorragique mesurant 5 cm aux dépens du col utérin sans extension aux parois vaginales ou aux paramètres et sans adénopathies inguinales palpables. Une biopsie a été alors réalisée et a montré un carcinome épidermoïde moyennement différencié invasif.

Une IRM pelvienne a été réalisée révélant un processus lésionnel tissulaire du col utérin de 55 mm sans extension locorégionale. Au total, il s'agissait d'un carcinome épidermoïde moyennement différencié invasif du col utérin stade IB 2 selon la Fédération internationale de gynécologie obstétrique (FIGO). La patiente a reçu une radiothérapie pelvienne à la dose de 46 Gy associée à une chimiothérapie concomitante à base de cisplatine suivie d'une curiethérapie utéro-vaginale à la dose de 20 Gy et d'une adénocolpohysterectomie sans conservation annexielle avec une lymphadénectomie pelvienne bilatérale. L'étude anatomopathologique de la pièce opératoire retrouvait des remaniements fibro-inflammatoires du col utérin sans prolifération résiduelle. Les paramètres, la collerette vaginale et les ganglions lymphatiques étaient indemnes. La patiente a été régulièrement suivie en consultation. En Juin 2011, la patiente a consulté, suite à l'apparition d'adénopathies sus-claviculaires bilatérales.

**Figure 1:**
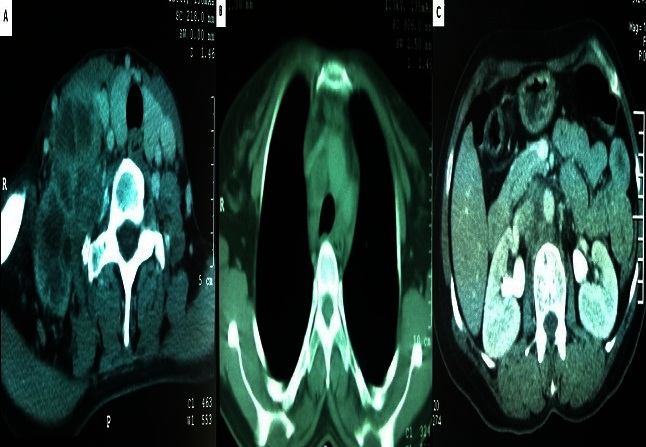
TDM thoraco-abdominale montrant des adénopathies sus-claviculaires bilatérales (A), para trachéales droites (B) et lombo-aortiques (C)

L'examen clinique retrouvait un magma d'adénopathies du creux sus claviculaire droit de 70 x 50 mm fixé au plan sous-jacent et une adénopathie du creux sus claviculaire gauche de 15 mm mobile et indolore. Le reste de l′examen somatique, était sans anomalies. Une tomodensitométrie cervico-thoraco-abdominopelvienne a montré des adénopathies cervicales sus claviculaires bilatérales, médiastinales et lombo-aortiques (Figure 1). Il n′y n'avait aucun autre site métastatique, notamment dans le foie et les poumons.

Une biopsie de l'adénopathie sus-claviculaire droite a été réalisée. L'examen anatomopathologique avec une étude immuno-histochimique a conclu à une métastase ganglionnaire d'un carcinome épidermoïde. La patiente a reçu une cure de chimiothérapie associant Paclitaxel et cisplatine.

**Figure 2:**
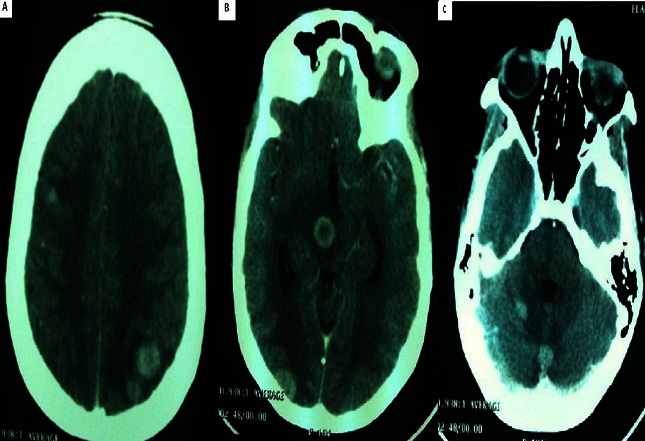
TDM cérébrale (coupe axiale) objectivant des métastases supra-tentorielles (A, B) et Cérébelleuses (C)

L'évolution était marquée par l'apparition brutale d'un syndrome d'hypertension intracrânienne, d'une baisse de l'acuité visuelle bilatérale et d'une diplopie. L'examen retrouvait un strabisme convergent de l'il gauche sans déficit sensitif ou moteur. Le scanner cérébral a mis en évidence au moins sept lésions nodulaires sus et sous-tentorielles prenant le contraste et entourées d'dème péri-lésionnel en faveur de localisations secondaires (Figure 2). La patiente a reçu une radiothérapie encéphalique totale à la dose de 20 Gy (5 fractions de 4 Gy). Devant l'altération rapide de l'état général, la patiente a été mise sous un traitement symptomatique et des soins de support.

## Discussion

Les métastases cérébrales à partir d'un cancer du col utérin sont extrêmement rares. Elles représentent 0,5 à 1,2% de toutes les métastases cérébrales [3–5]. Cette incidence ne cesse d'augmenter ces dernières années, en raison des avancées réalisées en matière de diagnostic et de traitement, aboutissant à une meilleure survie des patientes atteintes de cancer du col utérin [6]. L'allongement de la survie, permettrait la croissance des cellules métastatiques sanctuarisées dans l'encéphale [1].

En 1949, Henrisken [7] a décrit le premier cas de métastase cérébrale issue d'un cancer du col utérin sur une étude autopsique. Peu de cas ont été publiés depuis lors. Ces métastases surviennent habituellement dans la sixième décennie. [5, 8]. Par contre, notre patiente était d'un âge beaucoup plus jeune (44 ans), soit 16 ans de moins par rapport à la majorité des patientes rapportées dans la littérature.

La dissémination métastatique au cerveau d′un cancer du col de l′utérus se ferait par voie hématogène [8]. Le système veineux vertébral constitue la principale voie de ces métastases [4]. Cette dissémination dépend de la réponse immunitaire de la patiente, de la néovascularisation cérébrale, du nombre d′emboles vasculaires et des caractéristiques de la tumeur [9].

Ces métastases sont plus fréquemment observées avec des tumeurs du col utérin peu différenciées [3, 9, 10]. Dans notre cas, il s'agissait d'un carcinome épidermoide moyennement différencié. L′intervalle entre le diagnostic initial de cancer du col de l′utérus et la survenue de métastases cérébrales est considérablement variable, allant de 8 semaines à 8 ans [11]. Dadlani et al. ont rapporté l'observation d'une patiente ayant développée une métastase cérébelleuse solitaire 11 ans après le diagnostic d'un adénocarcinome du col utérin [12]. Une moyenne de 28,4 mois a été retrouvée par Ikeda et al [3] et de 18 mois par Cormio et al. [5]. Aucune relation entre le type histologique et l′intervalle de temps n′a jamais été établi [3, 5, 8, 10, 11]. Le diagnostic de métastase cérébrale chez notre patiente a été fait 14 mois après le diagnostic du cancer primitif.

La symptomatologie dépend du siège de la lésion et de l'existence de l'dème cérébral [8]. Les céphalées et l'hémiparésie sont les symptômes les plus fréquemment rapportés [2, 3, 5]. Les nausées, les vomissements, les troubles de la marche peuvent aussi exister et doivent alerter le clinicien [3].

Le diagnostic topographique repose essentiellement sur l′imagerie par résonance magnétique. Plus de 80% des métastases cérébrales sont situés dans la région sus-tentorielle du cerveau. Cette particularité pourrait être liée à la vascularisation et aux caractéristiques spatiales de cette région [5, 8, 9, 10]. Dans un tiers des cas, les lésions sont uniques et siègent fréquemment dans le lobe frontal [6, 13]. Dans notre cas, les lésions étaient multiples et situées à la fois dans la région sus et sous-tentorielle.

Les métastases cérébrales de cancer du col utérin surviennent généralement à un stade avancé de la maladie [8, 13] et dans le cadre de néoplasie polymétastatique, notamment dans les poumons, le foie et l'os [3, 4]. Les métastases pulmonaires semblent être les plus souvent associées [10]. Cependant, l'atteinte métastatique cérébrale isolée a été déjà décrite [5, 12]. Notre patiente présentait des métastases cérébrales associées à des métastases ganglionnaires lombo-aortique, médiastinale et sus claviculaire.

Il n'existe pas de standard thérapeutique des métastases cérébrales issues de cancers du col utérin. Le traitement dépend du nombre et du siège des lésions, du statut extracrânien de la maladie et de l'état général de la patiente (Indice de Karnofsky) [14].

Historiquement, les traitements des métastases cérébrales ont été basés sur la corticothérapie puis sur l'irradiation de l'encéphale en totalité [1]. La chirurgie est devenue un traitement local efficace [14]. Son indication reste indiscutable quand le pronostic vital est rapidement engagé (hémorragie, hydrocéphalie) et permet aussi d'obtenir le diagnostic anatomopathologique de certitude [1]. Elle constitue la meilleure option thérapeutique en cas de lésion unique ou de lésions multiples accessibles par la même voie d'abord de crâniotomie, en l'absence d'autres métastases à distance [3, 5, 6]. Cette chirurgie combinée à une radiothérapie adjuvante aboutit à une meilleure survie par rapport à la radiothérapie seule [2, 3, 5, 6, 10]. Par contre en cas de lésions multiples ou inopérables, la radiothérapie encéphalique totale est préconisé à visée palliative [10, 14].

La radiochirurgie et la radiothérapie stéréotaxique hypofractionnée sont actuellement des traitements reconnus des métastases cérébrales et peuvent être utilisées chez les patients porteurs de lésions petites et inaccessibles [9, 10].

La chimiothérapie à base de cisplatine joue un rôle important dans le traitement du cancer du col de l′utérus. Bien que son efficacité sur les métastases cérébrales soit encore inconnue [5], la chimiothérapie est le traitement de choix chez les patientes porteuses de métastases cérébrales multiples associées à des métastases extra-crâniennes [3, 6, 14].

Le pronostic de métastases cérébrales des cancers du col utérin dépend de l'âge des patientes, de l'état neurologique, du sous type histologique, de l'histoire clinique, du nombre des lésions et des comorbidités [13]. Il reste néanmoins sombre malgré toutes les options thérapeutiques [8, 13, 14] avec une survie moyenne très courte, de quelques mois seulement [2, 3, 6]. Cependant, un âge inférieur à 50 ans, un bon état général, une lésion cérébrale unique sans d'autres métastases extra-crâniennes sont liés un pronostic assez favorable [14].

## Conclusion

Les métastases cérébrales des cancers du col utérin sont extrêmement rares, possibles même après plusieurs années de rémission. Globalement de pronostic sévère, leur prise en charge dépend du nombre et du siège des lésions, du statut extracrânien de la maladie et de l'état général de la patiente. Elle nécessite une approche multimodale, en associant traitements locaux ciblés, comme la chirurgie et la radiothérapie stéréotaxique chez des patients sélectionnés, ainsi que des traitements systémiques.

## References

[1] Noël G, Daisne JF, Thillays F (2012). Radiothérapie en conditions stéréotaxiques des métastases cérébrales. Cancer Radiother.

[2] Amita M, Sudeep G, Rekha W, Yogesh K, Hemant T (2005). Brain metastasis from cervical carcinoma: a case report. MedGenMed..

[3] Ikeda SI, Yamada T, Katsumata N, Hida K, Tanemura K, Tsunematu R (1998). Cerebral metastasis in patients with uterine cervical cancer. Jpn J Clin Oncol..

[4] Chura JC, Shukla K, Argenta PA (2007). Brain metastasis from cervical carcinoma. Int J Gynecol Cancer..

[5] Cormio G, Pellegrino A, Landoni F, Regallo M (1996). Brain metastases from cervical carcinoma. Tumori..

[6] Lefkowitz D, Asconape J, Biller J (1983). Intracranial metastases from carcinoma of the cervix. South Med J..

[7] Henriksen E (1949). The lymphatic spread of carcinoma of the cervix and of the body of the uterus. A study of 420 necropsies. Am J Obstet Gynecol..

[8] Agrawal A, Kumar A, Sinha AK, Kumar M, Pandey SR, Khaniya S (2007). Intracranial metastases from carcinoma of the cervix. Singapore Med J..

[9] Weed JC, Graff AT, Shoup B, Tawfik O (2003). Small cell undifferentiated (neuroendocrine) carcinoma of the uterine cervix. J Am Coll Surg..

[10] Park SH, Ro DY, Park BJ, Kim YW, Kim TE, Jung JK (2010). Brain metastasis from uterine cervical cancer. J Obstet Gynaecol Res.

[11] Peters P, Bandi H, Efendy J, Perez-Smith A, Olson S (2010). Rapid growth of cervical cancer metastasis in the brain. J Clin Neurosci.

[12] Dadlani R, Ghosal N, Hegde AS (2012). Solitary cerebellous metastasis after prolonged remission in a case of uterine cervical adenocarcinoma. J Neurosci Rural Pract..

[13] Cordeiro JG, Prevedello DM, Da Silva Ditzel LF, Pereira CU, Araujo JC (2006). Cerebral metastasis of cervical uterine cancer: report of three cases. Arq Neuropsiquiatr..

[14] Hwang JH, Yoo HJ, Lim MC, Seo SS, Kang S, Kim JY (2013). Brain metastasis in patients with uterine cervical cancer. J Obstet Gynaecol Res.

